# Higher Renal Net Acid Excretion, but Not Higher Phosphate Excretion, during Childhood and Adolescence Associates with the Circulating Renal Tubular Injury Marker Interleukin-18 in Adulthood

**DOI:** 10.3390/ijms25031408

**Published:** 2024-01-24

**Authors:** Seyedeh-Masomeh Derakhshandeh-Rishehri, Luciana Peixoto Franco, Yifan Hua, Christian Herder, Hermann Kalhoff, Lynda A. Frassetto, Stefan A. Wudy, Thomas Remer

**Affiliations:** 1 DONALD Study Center, Department of Nutritional Epidemiology, Institute of Nutrition and Food Science, University of Bonn, 44225 Dortmund, Germany; 2 Institute for Clinical Diabetology, German Diabetes Center, Leibniz Center for Diabetes Research at Heinrich Heine University Düsseldorf, 40225 Düsseldorf, Germany; 3 German Center for Diabetes Research (DZD), 85764 München-Neuherberg, Germany; 4 Department of Endocrinology and Diabetology, Medical Faculty and University Hospital Düsseldorf, Heinrich Heine University Düsseldorf, 40225 Düsseldorf, Germany; 5 Research Department of Child Nutrition, St. Josef-Hospital, University Hospital of Pediatrics and Adolescent Medicine, Ruhr-University Bochum, 44791 Bochum, Germany; 6 Pediatric Clinic Dortmund, 44137 Dortmund, Germany; 7 Department of Medicine, Division of Nephrology, University of California San Francisco, San Francisco, CA 94143, USA; 8 Pediatric Endocrinology and Diabetology, Center of Child and Adolescent Medicine, Justus Liebig University, 35392 Giessen, Germany

**Keywords:** acid–base, ammoniagenesis, pro-inflammatory system, interleukin-18, phosphorus-intake, renal inflammation

## Abstract

High dietary phosphorus intake (P-In) and high acid loads may adversely affect kidney function. In animal models, excessive phosphorus intake causes renal injury, which, in humans, is also inducible by chronic metabolic acidosis. We thus examined whether habitually high P-In and endogenous acid production during childhood and adolescence may be early indicators of incipient renal inflammatory processes later in adulthood. P-In and acid–base status were longitudinally and exclusively determined by biomarker-based assessment in 277 healthy children, utilizing phosphate and net acid excretion (NAE) measurements in 24 h urine samples repeatedly collected between the ages of 3 and 17 years. Standard deviation scores (by sex and age) were calculated for anthropometric data and for the urinary biomarkers available within age range 3–17 years. Multivariable linear regression was used to analyze the relations of phosphate excretion and NAE with the adulthood outcome circulating interleukin-18 (IL-18), a marker of inflammation and kidney dysfunction. After adjusting for growth- and adulthood-related covariates and pro-inflammatory biomarkers to rule out confounding by non-renal inflammatory processes, regression models revealed a significant positive relationship of long-term NAE (*p* = 0.01), but not of long-term phosphate excretion with adult serum IL-18. Similar significant positive regression results were obtained after replacing NAE with 24 h urinary ammonium excretion as the exposition variable. Our results suggest that even moderate elevations in renal ammonia production, as caused by habitually higher acid loading during growth, may affect the intrarenal pro-inflammatory system in the long-term, known to be boosted by acidosis-induced raised ammoniagenesis.

## 1. Introduction

Interleukin-18 (IL-18) is a pro-inflammatory cytokine that had been initially identified as an interferon-γ-inducing factor. Alone or synergistically with other inflammasome-derived molecules, IL-18 induces the production of further potent pro-inflammatory cytokines and boosts natural killer cell activity [1,2]. IL-18 is produced by numerous organs and cell types and thus is involved in various inflammatory pathologies [1].

One important secretory source of IL-18 are the tubular epithelial cells of the kidney [1]. In line herewith, numerous diseases of the kidney demonstrate inflammatory processes and fibrosis [3]. Accordingly, elevated serum levels of IL-18 have been identified as potential predictors of renal dysfunction, fibrosis, and progression of nephropathy in patients with type 2 diabetes [4,5]. Additionally, a high urinary excretion of IL-18 has been shown to be an early diagnostic biomarker of kidney dysfunction and acute kidney injury [2,6]. 

One cause of kidney-related inflammatory processes is acidosis. Acidosis-induced elevation of intrarenal ammoniagenesis can, partly via complement activation [7], stimulate secretion of pro-inflammatory cytokines including IL-1β and IL-18 [8,9,10].

While there are convincing patho-metabolic data indicating that pronounced metabolic acidosis-stimulated increases in local ammonia concentration can trigger maladaptive complement activation with increased inflammatory and pro-fibrotic responses [9,11], there exist no data on whether milder increases in endogenous acid production over long periods of time may go along with already distinct signs of renal inflammation.

We hypothesized that in people with a consistently higher renal acid load during childhood and adolescence, one of the major biomarkers of renal inflammatory processes, namely the tubular injury marker IL-18, could indicate some degree of renal function worsening. A high nutritional phosphorus intake, which regularly accompanies increased acid loads in the diet, may also negatively affect kidney function. Therefore, we examined the long-term associations of both a higher renal NAE and a higher 24 h urinary phosphate excretion during childhood and adolescence with the circulating IL-18 concentration in later adulthood. 

## 2. Results

### 2.1. Participants’ Characteristics

The anthropometric, blood, and 24 h urinary characteristics of the participants are presented longitudinally in Table 1. The mean age of 277 subjects (134 males) was 4 years at the first assessment and 17 years at the last assessment. Their 24 h urinary pH fluctuated around 6.4 with a modestly lower mean value (*p* = 0.02) at the age of last assessment. Within this age period, absolute values of NAE and PO_4_ excretion increased markedly by 30.2 mEq/d and 13.3 mmol/d, respectively (*p <* 0.0001). Anthropometric parameters and the other 24 h urinary excretion rates showed the expected significantly higher values at the last assessment (*p <* 0.0001). Estimates of phosphorus and protein intakes exceeded the respective dietary recommendations [12,13] in more than half of the children and adolescents (Table 1); furthermore, these biomarker-based findings corresponded closely to recent dietary recordings in DONALD probands of comparable age [14].

### 2.2. Longitudinal Prospective Association of NAE and Phosphate Excretion during Growth with Adulthood IL-18 

The results of multiple linear regression analyses for the examination of the relationships of acid–base status and biomarker-based assessed P-In during growth with circulating IL-18 in adulthood are shown in Table 2. After adjustment for sex, adult age, nutrition-related urinary biomarkers, and adult blood parameters, a significant positive association was observed between long-term net endogenous acid production during childhood and adolescence and circulating IL-18 in adulthood (*β* = 54.0; *p =* 0.02 [model II]). The effect strength of this association did not change in the final model after additional adjustment for biomarkers of inflammation. An absence of significant association was observed between the marker of long-term P-In during growth and circulating IL-18 during adulthood. 

The result seen for the NAE-IL-18 relationship was further confirmed by a third analysis using the more renal ammonia production-specific parameter ammonium excretion as the exposition variable instead of NAE (Table 2). Also, the higher renal ammonia production during the growing years was significantly related to adult blood concentrations of IL-18 in the fully adjusted model. 

To separately assess the relationship of phosphorus intake and NAE during growth with two other kidney health-relevant parameters, i.e., GFR and HOMA-IR, two separate multiple regressions with the latter blood measurement-derived parameters as outcomes were run (Supplemental Table S1). Neither young adulthood GFR nor HOMA-IR showed a significant association with long-term pre-adulthood net endogenous acid production or phosphate excretion (Supplemental Table S1).

## 3. Discussion

Our current longitudinal analysis covering the age range from childhood to young adulthood provides prospective evidence that a higher net endogenous acid production during the growing years may be involved in increased kidney tubular inflammatory processes in later years. This finding was substantiated by demonstrating that higher renal ammonium production during growth—biomarker-based assessed via repeated measurements of 24 h urinary ammonium excretion—was significantly associated with circulating levels of the tubular injury marker IL-18 in adulthood. However, a habitually high dietary phosphorus intake, which may potentially negatively impact kidney function [18,19], did not reveal a prospective relationship with adult serum levels of IL-18.

Although direct comparisons with similar studies in the field of acid–base research are not possible, as there are no examinations on healthy individuals’ habitual acid–base status and potential long-term inflammatory responses are available, several studies in different kinds of patients corroborate the present findings. Accordingly, in children suffering from diabetic ketoacidosis [20,21,22], IL-18 has been shown to be positively associated with their acidotic state. Raised levels of inflammatory biomarkers have also been reported in milder forms of acidosis with only moderately reduced serum bicarbonate concentrations [23,24,25]. Among numerous studies showing higher inflammatory responses with acidotic states, only one in vitro study applying hydrochloric and lactic acid to a cell culture system found inconsistent results [26]. In particular, various clinical research studies in patients with chronic kidney disease and metabolic acidosis have revealed that the prolonged upregulation of the kidney’s major acid excretion mechanism, i.e., an elevated ammoniagenesis, promotes inflammation and disease progression [7,27,28,29]. 

However, ammoniagenesis needs to be stimulated with ensuing NAE rises if increased amounts of acid equivalents are endogenously produced, e.g., due to a regular consumption of rather animal protein-rich diets with a low fruit and vegetable (F&V) content. [30] These diets provide an excess of acid anions (e.g., sulfate, phosphate, chloride, unmetabolizable organic acids) compared to alkali cations. The kidneys’ provision of additional NH_4_^+^ allows for excretion of surplus acid anions without losing too much alkali, i.e., bicarbonate. 

Accordingly, renal ammoniagenesis is an important physiologically process that accounts for the majority of basal bicarbonate regeneration [31]. As the major source of renal ammonia generation are amino acids, and in particular glutamine [31], temporary increases in acid loads, e.g., due to elevated protein intakes, are physiologically balanced by a raised ammonia production along with an abundant glutamine availability. Correspondingly, ammonium generation drastically falls with dietary protein restriction [32], and for a given protein intake level it decreases too if the consumption of F&V or alkali salts rises [33]. 

**Figure 1:**
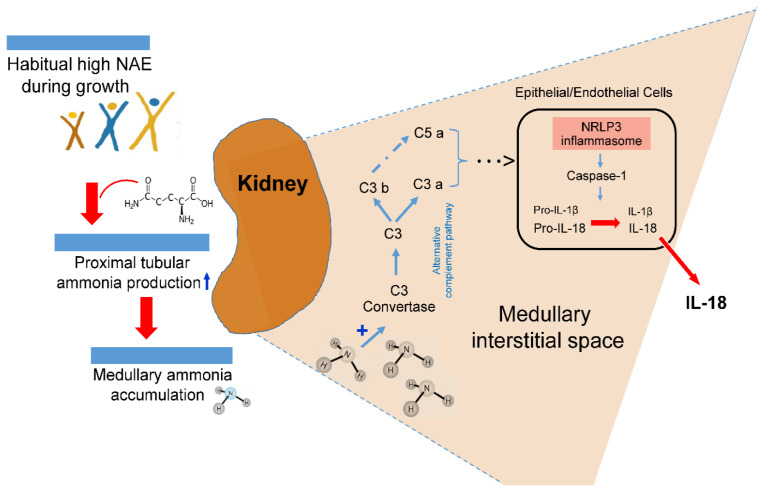
Suggested possible intrarenal mechanism of inflammasome activation boosted by acidosis-induced elevated ammonia production.

The major patho-biological mechanism behind the potential NAE–renal inflammation relationship, observed in the present study, may be ascribed to the probable tissue toxicity of chronically elevated ammonia concentrations [7,27]. In previous tissue and animal studies, augmented intrarenal ammonia levels have been found to be associated with increased tubulo-interstitial injury [9]. Different research groups have provided experimental evidence that the toxic and proinflammatory effects of elevated intrarenal ammonia concentrations can induce tubulo-interstitial inflammation processes, mediated via activation of the alternative complement pathway [7,9,34]. A possible mechanism of a high acid load-induced elevation of renal inflammatory processes is sketched in Figure 1.

In patients with chronic tubulointerstitial nephritis, non-immune metabolic noxae, such as hypokalemia, hyperoxaluria, and hyperuricemia, are involved, along with a medullary ammonia accumulation in the activation of the kidney’s tubular and interstitial cells’ inflammasome complex that induces maturation of pro-inflammatory IL-1β and IL-18 [10]. In mouse models of kidney-related injury and inflammation caused by dietary potassium deficiency, Terker et al. [8] demonstrated that those particular mouse knockout models which led to a reduction in the ammoniagenesis pathway did also prevent kidney injury and inflammation. The slowing of chronic kidney disease progression through a reduction in ammonia generation [7], i.e., a reduction in chronic acid stress by regular alkali treatment or an appropriate nutrition with a low potential renal acid load, has been put into focus by Wesson, Goraya, and colleagues [25,35,36,37]. Our present findings add to this concept of acid stress and provide epidemiological evidence that the reduction of an already higher endogenous acid production related to normal protein-rich and rather low-F&V diets may in the long-term compromise kidney health. A reduction in the relatively high intake of acid-forming dairy, grains, and egg and meat products of many DONALD children and adolescents [14] and an increase in their F&V consumption could be an important preventive measure not only to reduce the participants’ potential incipient renal inflammatory processes, but also the long term risks for various kidney related diseases [25,37].

### Study Limitations and Strengths

One of the limitations of this study is that—using the urinary biomarker of phosphate excretion—we could not differentiate between intakes of natural phosphorus contained in food and phosphorus added to food, as has recently been performed based on food frequency questionnaire data [18]. A study using population-based data of the Jackson Heart Study revealed negative effects on kidney function specifically for added, but not for total phosphorus from natural sources. Hence, possible renal inflammatory effects, particularly of habitual higher added phosphorus intakes, cannot be excluded.

Also, the sample size of 277 individuals may not have been high enough to identify at least a trend in a possible relationship between long term biomarker-based assessed phosphorus intake and incipient inflammatory processes, although average total phosphorus intake was at a rather high level.

Additionally, it has to be mentioned that the present results obtained in a cohort of Caucasian children are not necessarily applicable to other ethnicities.

Due to the fully noninvasive characteristic during the whole growth period of the DONALD study, we were also not able to assess the baseline inflammatory state of the study participants during childhood or adolescence.

On the other hand, it has to be emphasized that we could examine the long-term acid base status of healthy individuals over their growing years by using the gold standard not only for measurement of net endogenous acid production, but also for the non-invasive determination of renal ammonia production, of which ~50% is excreted in the urine [38].

In addition, it was possible to check and adjust for protein intake (biomarker-based) and relevant kidney-function related factors, such as HOMA-IR, circulating uric acid, and GFR during adulthood. 

Finally, as circulating IL-18 levels can also be increased due to inflammatory processes in the body, independently of kidney function, the consideration and inclusion of major additional, not specifically kidney-related, markers of inflammation in our analysis allowed us to rule out confounding by non-renal inflammatory foci. 

## 4. Materials and Methods

### 4.1. Study Population

Healthy children and adolescents aged 3–17 years were selected from the ongoing Dortmund Nutritional and Anthropometric Longitudinally Designed (DONALD) study. The DONALD study is an open-cohort study which started in 1985 and collects information on diet, metabolism, and growth in healthy volunteers from childhood until adulthood. For children from 3 to 4 y onward, anthropometric measurements, a 3D weighed dietary record, 24 h urine collections, and medical examinations as well as interviews on lifestyle such as health, behavior, and environment are scheduled in yearly intervals. All assessments were performed with the written consent from parents and grown-up children, and the study protocol was approved by the ethics committee of the university.

**Figure 2:**
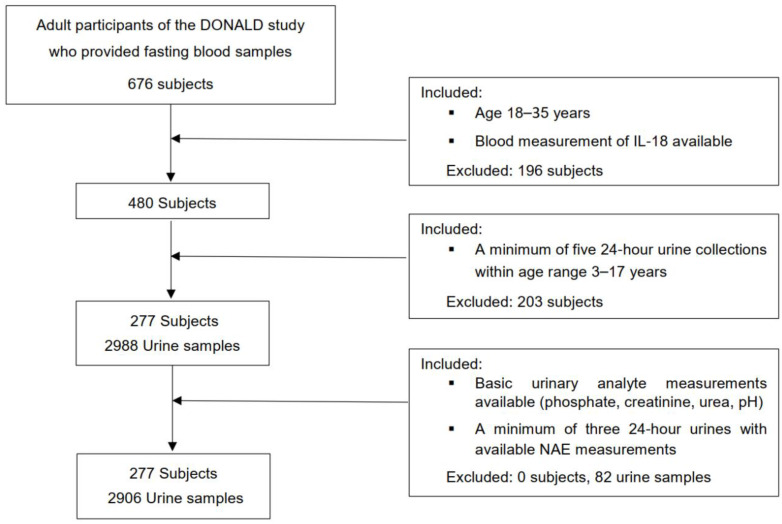
Flowchart of the present study.

For this exclusively biomarker-based examination, an initial number of 573 participants of the DONALD study were considered, in whom a minimum of 5 eligible 24 h urine collections were available during growth, at least 2 within childhood (3–8 y) and at least 3 within adolescence (9–17 y). Those participants who had at least five 24 h urines with completed measurements of urinary pH, PO_4_, creatinine, and urea nitrogen, and a minimum of three 24 h urines with completed NAE measurements between age 3 and 17 years were further selected (n = 521). Finally, out of 521 participants, 277 individuals could be included in whom, also during adulthood (18–35 y), measurements of circulating IL-18 had been performed (Figure 1). In the case of more than one accomplished IL-18 blood measurements of participants (n = 41) being available, the respective IL-18 value of the last blood collection within age range 18 to 35 years was selected (Figure 2). 

### 4.2. Anthropometric Measurements

Anthropometric measurements of the study population were taken by trained nurses based on standardized procedures. Body weight was determined to the nearest 0.1 kg with a digital scale (Seca 753E; Seca Weighing and Measuring Systems, Hamburg, Germany), and standing height to the nearest 0.1 cm with a stadiometer (Harpenden, Holtain Ltd., Crymych, UK). Body mass index (BMI) was calculated from the following equation: weight/height^2^ (kg/m^2^). 

### 4.3. Urinary Measurements

Personal and written instructions were given to each child and their parents on how to collect the 24 h urine sample at home using preservative free, Extran-cleaned (Extran, MA03; Merck, Darmstadt, Germany) 1-L plastic containers. The urine samples were stored at −18 to −20 °C until they were thawed for analysis. Creatinine excretion was quantified with a creatinine analyzer (Beckman-2; Beckman Instruments, Fullerton, CA, USA) based on the kinetic Jaffe’ procedure. To minimize possible errors in urine collection, samples with a daily creatinine excretion < 0.1 mmol/kg were not considered in the analysis [39]. Urinary urea was measured using the urease Berthelot method (Randox Laboratories, Crumlin, UK). Acid–base analytes, i.e., 24 h pH, titratable acidity (mEq/L), ammonium (mmol/L), and bicarbonate (mmol/L) were quantified by the three phase acid–base titration method [40], using a Mettler Toledo endpoint titrator (Mettler Toledo, Giessen, Germany), and NAE was then calculated by summing titratable acid and ammonium minus bicarbonate. Urinary phosphate excretion was determined with a Dionex 2000i/SP ion chromatograph that contained an ion Pac AS4A column (Dionex GmbH, Idstein, Germany).

### 4.4. Blood Measurements

After an overnight fast, venous blood samples (<20 mL) were collected, centrifuged at 4 °C within 15 min, and stored at −80 °C. Serum IL-18 (Human IL-18 ELISA, MBL, Nagoya, Japan) and serum or plasma concentrations of 10 other biomarkers of inflammation were measured at the Institute for Clinical Diabetology (German Diabetes Center, Düsseldorf, Germany) using highly sensitive ELISAs as previously described (leptin, adiponectin, soluble intercellular adhesion molecule-1 (sICAM-1), omentin, IL-1 receptor antagonist (IL-1RA), chemerin, fetuin-A, IL-6, fibroblast growth factor-21 (FGF21), soluble Eselectin (sE-selectin)) [41]. Plasma glucose, LDL and HDL cholesterol, as well as uric acid were determined at the clinic lab of the pediatric clinic Dortmund, Germany, and high-sensitivity C-reactive protein (hsCRP) at the lab of Institute for Clinical Diabetology with a Roche/Hitachi Cobas c311 analyser (Roche diagnostics, Mannheim, Germany). 

Plasma insulin concentration was measured at the Laboratory for Translational Hormone Analytics of the University of Giessen with an immunoradiometric assay (IRMA; DRG Diagnostics, Marburg, Germany) and the homeostasis model assessment was used to determine insulin resistance (HOMA-IR) [42]. GFR was estimated using the new creatinine-based equations according to the Chronic Kidney Disease Epidemiology Collaboration [43].

### 4.5. Statistical Analysis

For all statistical analyses, SAS statistical software (SAS Institute Inc., Cary, NC, USA; version 9.2) was used, and *p*-values < 0.05 were considered significant. Normal distribution of all variables were checked using the Shapiro–Wilk test and Q–Q plot. General characteristics of the participants are presented as mean (±SD) for normally distributed parameters and as median (25th, 75th percentiles) if not normally distributed. Differences between the first and the last assessment were tested using the paired t-test for normally distributed characteristics and the Wilcoxon signed-rank test for non-normally distributed ones. All 24 h urinary biomarkers and anthropometric parameters were internally standardized (mean = 0, SD = 1) by sex and age group, and the respective standardized deviation scores (SDS) were averaged for each person. Childrens’ and adolescents’ anthropometry, diet, and acid–base-related data were included in the analyses as individual arithmetic means of SDS. 

Multiple linear regression models (PROC GLM) were used to examine the prospective associations of long-term exposure to higher net endogenous acid production (NAE) or higher phosphorus intake (PO_4_ excretion) in childhood and adolescence with circulating IL-18 in adulthood as outcome. Since sex-specific interactions were not observed (*p >* 0.1), all regression analyses were conducted without sex stratification. Before analyses, all 4 assumptions for multi-linear regressions, i.e., normal distribution of residuals, linearity, non-multicollinearity, and homoscedasticity, were checked and not violated. Each potential covariate was tested separately and stepwise in the regression models and remained included if: (i) it markedly modified the association between the exposition variable NAE or PO_4_ excretion and the outcome IL-18 (i.e., if changes in β coefficient of the exposition variable were ≥10%), (ii) it had an independent and significant fixed effect on the outcome IL-18 (*p* < 0.05), or (iii) it improved the explained variability of the whole model.

A three-step regression model was used and adjusted for the following covariates: sex, urinary urea-nitrogen excretion, urinary pH, adult age, and adults’ circulating levels of hsCRP and uric acid, as well as HOMA-IR, GFR, and LDL/HDL ratio. Moreover, to specifically exclude contributions of inflammatory processes other than those of renal origin to the variation in IL-18, ten further biomarkers of inflammation were checked regarding their relationships with the study outcome. Of those 10 inflammatory markers, only 3 showed significant correlations with IL-18 (sICAM1, r = 0.36, *p* < 0.0001; sE-selectin, r = 0.26, *p* < 0.0001; IL-1RA, r = 0.28, *p* < 0.0001). Those 3 were included in the final model in 2 steps (pairwise first: sE-selectin and IL-1RA, showing no co-correlation with each other; then: sICAM1 with co-correlation with both other biomarkers of inflammation). This additional adjustment increased the model’s R^2^ further without any reduction in the β value for NAE. To particularly specify the potential relevance of the ammonium component of NAE for the outcome, the regression models were run again with 24 h ammonium excretion as the exposition variable instead of NAE. Possible associations of NAE and PO4 excretion as predictors with GFR and HOMA-IR as additional outcomes were also examined and are shown in Supplementary Table S1. 

## 5. Conclusions

In conclusion, a habitually higher net endogenous acid production during childhood and adolescence may enhance—apparently biologically mediated via raised intrarenal ammoniagenesis—later inflammatory events in the adult kidney. 

Overall, the present results suggest that a habitually higher need to renally excrete surplus protons, i.e., an increased ammoniagenesis over a long time period, although still within the physiological range, may already contribute to pro-inflammatory renal processes and, thus, may indicate commencing renal acid stress.

These findings underpin the importance of long-term clinically relevant nutritional advice and dietary recommendations to promote metabolically alkalizing F&V-rich, low-animal-protein diets already from early childhood onward in order to create a preventative basis for sustained kidney and overall health. 

## Figures and Tables

**Table 1:** Characteristics of participants during growth and adulthood ^a^.

Childhood and Adolescence	Longitudinal Overview ^b^	
	First Assessment	Last Assessment ^c^
n (male/female)	277 (134/143)	277 (134/143)
Age, y	4.0 (3.4, 5.0)	17.0 (15.3, 17.0)
Weight, kg	17.4 (15.6, 19.6)	64.6 ± 13.0
Height, cm	105.6 (100.8, 111.8)	172.9 ± 10.4
BMI, kg/m^2^	15.5 (14.8, 16.4)	21.1 (19.4, 23.1)
Urine pH	6.4 ± 0.5	6.3 ± 0.5
NAE, mEq/d	23.5 ± 12.0	53.7 ± 26.2
Ammonium, mmol/d	19.0 ± 5.8	42.1 ± 14.5
TA, mEq/d	8.5 ± 4.9	18.9 ± 10.2
PO_4_, mmol/d	13.3 ± 4.1	26.6 ± 8.6
Phosphorus intake (mg/d) ^d, e^	654.9 ± 201.9	1309.8 ± 423.5
Urea-N, mmol/d	158.4 (130.6, 193.8)	336.3 (271.1, 408.4)
Protein intake (g/d) ^f, g^	40.5 (35.6, 46.8)	71.9 (60.4, 84.7)
Creatinine, mmol/d	2.6 ± 0.7	11.7 ± 3.4
Adulthood		
Adults’ age, y	21.4 ± 3.9	
Glucose, mg/dL	92.4 ± 17	
Insulin, µIU/mL	12.6 ± 5.7	
HOMA-IR	1.6 ± 0.7	
GFR (mL/min/1.73 m^2^)	104.7 ± 19.5	
Uric acid (mg/dL)	5.3 ± 1.2	
IL-18, pg/mL	260.3 ± 95.8	
hsCRP, mg/dL	0.2 ± 0.7	
IL-1RA, pg/mL	257.5 ± 156.1	
sE-Selectin, ng/mL	33.4 ± 12.6	
sICAM1, ng/mL	193.8 ± 41.5	

Abbreviations: BMI, body mass index; FMI, fat mass index; NAE, net acid excretion; PO_4_, phosphate; Urea-N, 24 h urinary urea nitrogen excretion; TA, titrable acid; hsCRP, high-sensitivity C-reactive protein; HOMA-IR, homeostasis model assessment-insulin resistance; IL-1RA, interleukin-1 receptor antagonist; sE-selectin, soluble E-selectin; sICAM1, soluble intercellular adhesion molecule-1; GFR, glomerular filtration rate. ^a^ All values are means ± SDs if normally distributed or median (25th, 75th percentiles) if not normally distributed. ^b^ The first and the last urine sample of each participant who had at least 2 eligible urine samples collected between age 3 and 8 y, and at least 3 eligible urine samples collected between age 9–17 y (*n* = 277; average number of urines collected per person = 10.5). ^c^ Differences between the first and last assessments were tested with paired *t* test for normally distributed variables and Wilcoxon signed rank test for non-normally distributed ones (all differences *p* < 0.0001, except 24 h urinary pH). ^d^ Total phosphorus intake per day, calculated—biomarker-based—from 24 h urinary phosphate excretion (mmol/d) under the assumption of an average intestinal absorption rate of 63% [15] (multiplication of PO4 excretion by 1.59 and by 30.97 considering absorption losses and atomic weight of phosphorus, respectively). ^e^ Recommended dietary allowances for phosphorus for 4 years old children and 17 years old male and female adolescents: 500 mg/d and 1250 mg/d, respectively [12]. ^f^ Total protein intake per day, estimated-biomarker-based-from 24 h urinary urea nitrogen excretion. Values are means of the respective results of two clinical estimation formulas: (a) protein intake (g/day) = urea-N excretion (g/day) × 9.5 [16] and (b) protein intake (g/day) = (urea-N excretion (g/day) + 4) × 6.25 [17]. ^g^ Recommended dietary allowances for protein for 4 years old children and 17 years old male adolescents as well as 17 year old female adolescents: 19 g/d, 52 g/d, 46 g/d, respectively [13].

**Table 2:** Prospective relationship of biomarkers of net endogenous acid production and phosphorus intake during growth with circulating interleukin 18 (IL-18) in adulthood ^a^.

		Β (95% CI)	R^2^	*p*
NAE-SDS				
	Model I ^b^	40.64 (−5.46, 86.75)	0.03	0.08
	Model II ^c^	54.02 (7.27, 100.77)	0.11	0.02
	Model III ^d^	54.25 (11.28, 97.23)	0.26	0.01
PO4-SDS				
	Model I ^b^	23.47 (−9.68, 56.62)	0.02	0.16
	Model II ^c^	20.84 (−12.88, 54.56)	0.10	0.22
	Model III ^d^	8.32 (−23.14, 39.78)	0.24	0.60
NH4-SDS				
	Model I ^b^	17.13 (−10.73, 44.99)	0.02	0.23
	Model II ^c^	26.94 (−1.64, 55.52)	0.10	0.06
	Model III ^d^	30.93 (4.69, 57.16)	0.26	0.02

Abbreviations: NAE-SDS, PO4-SDS, and NH4-SDS; individual means of standard deviation scores of children’s and adolescents’ 24 h net acid excretion, 24 h phosphate excretion, and 24 h ammonium excretion. ^a^ Results obtained from step-wise multi linear regression analyses. ^b^ Model I adjusted for sex and adult age along with basic urinary excretion variables related to acid–base status: urea nitrogen and urinary pH. ^c^ Model II adjusted for variables in model I plus adults’ blood parameters: HOMA-IR, GFR, uric acid, LDL/HDL ratio, and CRP. ^d^ Model III adjusted for variables in model II plus circulating pro-inflammatory effect modifiers: IL-1RA, sE-Selectin, and sICAM1.

## Data Availability

Data described in the manuscript, code book and analytic code of this study will be made available upon request. DONALD study data are available upon reasonable request for research questions within the scope of the DONALD Study, which are consistent with the legal and ethical standard practices of the DONALD study.

## Supplementary Materials

The following supporting information can be downloaded at: https://www.mdpi.com/article/10.3390/ijms25031408/s1.
