# Correction: Derakhshandeh-Rishehri et al. Higher Renal Net Acid Excretion, but Not Higher Phosphate Excretion, During Childhood and Adolescence Associates with the Circulating Renal Tubular Injury Marker Interleukin-18 in Adulthood. *Int. J. Mol. Sci.* 2024, *25*, 1408

**DOI:** 10.3390/ijms27219581

**Published:** 2026-10-27

**Authors:** Seyedeh-Masomeh Derakhshandeh-Rishehri, Luciana Peixoto Franco, Yifan Hua, Christian Herder, Hermann Kalhoff, Lynda A. Frassetto, Stefan A. Wudy, Thomas Remer

**Affiliations:** 1 DONALD Study Center, Department of Nutritional Epidemiology, Institute of Nutrition and Food Science, University of Bonn, 44225 Dortmund, Germany; ma.derakhshandeh@gmail.com (S.-M.D.-R.); lfranco@uni-bonn.de (L.P.F.); yifan_hua@outlook.com (Y.H.); 2 Institute for Clinical Diabetology, German Diabetes Center, Leibniz Center for Diabetes Research at Heinrich Heine University Düsseldorf, 40225 Düsseldorf, Germany; christian.herder@ddz.de; 3 German Center for Diabetes Research (DZD), 85764 München-Neuherberg, Germany; 4 Department of Endocrinology and Diabetology, Medical Faculty and University Hospital Düsseldorf, Heinrich Heine University Düsseldorf, 40225 Düsseldorf, Germany; 5 Research Department of Child Nutrition, St. Josef-Hospital, University Hospital of Pediatrics and Adolescent Medicine, Ruhr-University Bochum, 44791 Bochum, Germany; hermann.kalhoff@klinikumdo.de; 6 Pediatric Clinic Dortmund, 44137 Dortmund, Germany; 7 Department of Medicine, Division of Nephrology, University of California San Francisco, San Francisco, CA 94143, USA; lynda.frassetto@ucsf.edu; 8 Pediatric Endocrinology and Diabetology, Center of Child and Adolescent Medicine, Justus Liebig University, 35392 Giessen, Germany; stefan.wudy@paediat.med.uni-giessen.de

The Original Investigation entitled “Higher Renal Net Acid Excretion, but Not Higher Phosphate Excretion, during Childhood and Adolescence Associates with the Circulating Renal Tubular Injury Marker Interleukin-18 in Adulthood” [1], published in *Int. J. Mol. Sci.* in 2024, has been corrected to fix an error in Supplementary Table S1 and the corresponding textual context in the Results section. Supplementary Table S1 showed an initial assessment of the prospective relationships of children’s and adolescents’ net endogenous acid production, measured as 24 h net acid excretion (NAE) and phosphorus intake with the outcomes glomerular filtration rate (GFR) and homeostasis model assessment-insulin resistance (HOMA-IR). In the case of the exposure habitual 24 h NAE (NAE-SDS), the children’s and youth’s 24 h urine pH was also inadvertently included in this data analysis as a covariate, resulting in a mistaken non-significant finding for the NAE–GFR relationship, whereas the 24 h urine pH was positively associated with GFR in the regression model.

Since 24 h urine pH and 24 h NAE are strongly correlated, means of repeated measurements of either the one or the other, but not of both—pH and NAE—together in the same model, are valid exposure markers to approximately characterize long-term average acid-base status in regression or correlation analyses. A recently published targeted examination assessing each exposure marker separately [2] strengthened the importance of habitual acid-base status during growth for the later GFR level in young adulthood. In the accordingly corrected Supplementary Table S1 not including 24 h urine pH (given below), a significant inverse association (*p* ≤ 0.01) of pre-adulthood average NAE with GFR is now discernible which is in line with the detailed and specific subsequent analyses [2]. In the Results section the corrected last sentence now should read: “Neither young adulthood GFR nor HOMA-IR showed a significant association with long-term pre-adulthood phosphate excretion; however, young adults’ GFR did significantly inversely associate with habitual pre-adulthood NAE in this preliminary examination (Supplemental Table S1—Corrected)”.

**Supplemental Table S1—Corrected:** Prospective relationship of biomarkers of net endogenous acid production and phosphorus intake during growth with GFR and HOMA-IR in adulthood ^a^.

		*Β*	*R* ^2^	*p*
		*(95% CI)*		
**GFR**				
**NAE-** **SDS**	Model I ^b^	−7.97 (−13.35, −2.59)	0.08	0.005
	Model II ^c^	−7.87 (−13.19, −2.56)	0.13	0.005
	Model III ^d^	−7.07 (−12.38, −1.75)	0.15	0.01
**PO4-** **SDS**	Model I ^b^	−2.61 (−9.14, 3.92)	0.10	0.43
	Model II ^c^	−2.32 (−8.82, 4.18)	0.14	0.48
	Model III ^d^	−1.91 (−8.44, 4.62)	0.16	0.56
**HOMA-IR**				
**NAE-** **SDS**	Model I ^b^	−0.04 (−0.24, 0.16)	0.03	0.70
	Model II ^c^	−0.05 (−0.26, 0.15)	0.08	0.62
	Model III ^d^	−0.05 (−0.26, 0.16)	0.09	0.63
**PO4-** **SDS**	Model I ^b^	0.04 (−0.20, 0.28)	0.03	0.74
	Model II ^c^	0.01 (−0.23, 0.26)	0.08	0.91
	Model III ^d^	0.01 (−0.24, 0.26)	0.08	0.95

Abbreviations: NAE-SDS and PO4-SDS; individual means of standard deviation scores of children’s and adolescents’ 24 h net acid excretion and 24 h phosphate excretion. HOMA-IR; homeostasis model assessment-insulin resistance. GFR; glomerular filtration rate. ^a^ Results obtained from step-wise multi linear regression analyses. ^b^ Model I adjusted for sex and adult age along with the basic urinary excretion variable related to acid-base status, i.e., urea nitrogen and in case of the exposure PO4-SDS additionally with the 24 h urine pH. ^c^ Model II adjusted for variables in model I plus adults’ blood parameters: uric acid, LDL/HDL ratio, CRP, and HOMA-IR (in case of outcome GFR) or GFR (in case of outcome HOMA- IR). ^d^ Model III adjusted for variables in model II plus circulating pro-inflammatory effect modifiers: IL-1RA, sE-Selectin, and sICAM1.

The authors state that the scientific conclusions regarding the circulating renal tubular injury marker interleukin-18 are unaffected.

All authors have read and agreed to the published version of the manuscript. This correction was approved by the Academic Editor. The original publication has also been updated.
