# Supplementary material for: Higher Renal Net Acid Excretion, but Not Higher Phosphate Excretion, during Childhood and Adolescence Associates with the Circulating Renal Tubular Injury Marker Interleukin-18 in Adulthood
Source: Int J Mol Sci. 2024 Jan 24;25(3):1408. doi: 10.3390/ijms25031408 (PMC10855358; doi:10.3390/ijms25031408)
Supplement: Supplementary file 1 [file ijms-25-01408-s001.zip › ijms-2788360-supplementary.pdf]

Supplementary table S1. Prospective relationship of biomarkers of net endogenous acid production and phosphorus intake during growth with GFR and HOMA-IR in adulthood [a]

			B *(95% CI)*	R^2	*P*
**GFR**					
	**NAE-SDS**				
		Model I [b]	0.43 (-8.62, 9.48)	0.10	0.92
		Model II [c]	-0.54 (-9.63, 8.56)	0.14	0.91
		Model III [d]	-0.51 (-9.56, 8.53)	0.16	0.91
	**PO4-SDS**				
		Model I [b]	-2.61 (-9.14, 3.92)	0.10	0.43
		Model II [c]	-2.32 (-8.82, 4.18)	0.14	0.48
		Model III [d]	-1.91 (-8.44, 4.62)	0.16	0.56
**HOMA-IR**					
	**NAE-SDS**				
		Model I [b]	-0.11 (-0.45, 0.23)	0.03	0.53
		Model II [c]	-0.07 (-0.41, 0.28)	0.08	0.70
		Model III [d]	-0.06 (-0.41, 0.28)	0.08	0.71
	**PO4-SDS**				
		Model I [b]	0.04 (-0.20, 0.28)	0.03	0.74
		Model II [c]	0.01 (-0.23, 0.26)	0.08	0.91
		Model III [d]	0.01 (-0.24, 0.26)	0.08	0.95

Abbreviations: NAE-SDS and PO4-SDS; individual means of standard deviation scores of children's and adolescents´ 24-h net acid excretion and 24-h phosphate excretion. HOMA-IR; homeostasis model assessment-insulin resistance. GFR; glomerular Filtration rate.

[a] Results obtained from step-wise multi linear regression analyses.

[b] Model I adjusted for sex and adult age along with basic urinary excretion variables related to acid-base status: urea nitrogen and urinary pH.

[c] Model II adjusted for variables in model I plus adults' blood parameters: uric acid, LDL/HDL ratio, CRP, and HOMA-IR (in case of outcome GFR) or GFR (in case of outcome HOMA-IR).

[d] Model III adjusted for variables in model II plus circulating pro-inflammatory effect modifiers: IL-1RA, sE-Selectin, and sICAM1.
